# Incidence and predictors of hemodynamic compromise due to high-grade AV block after TAVI

**DOI:** 10.3389/fcvm.2023.1161871

**Published:** 2023-06-06

**Authors:** Maren Weferling, Stefan Lan Cheong Wah, Ulrich Fischer-Rasokat, Andreas Hain, Matthias Renker, Efstratios I. Charitos, Christoph Liebetrau, Julia Treiber, Yeong-Hoon Choi, Christian W. Hamm, Won-Keun Kim

**Affiliations:** ^1^ Department of Cardiology, Kerckhoff Heart and Thorax Center, Bad Nauheim, Germany; ^2^ German Center for Cardiovascular Research (DZHK), Partner Site RheinMain, Bad Nauheim, Germany; ^3^ Department of Cardiac Surgery, Kerckhoff Heart and Thorax Center, Bad Nauheim, Germany; ^4^ Cardioangiological Center Bethanien (CCB), Department of Cardiology, Agaplesion Bethanien Hospital, Frankfurt, Germany; ^5^ Department of Cardiology, University Hospital of Giessen, Giessen, Germany

**Keywords:** AV block, cardiac arrest, cardiac resuscitation, pacemaker, TAVI

## Abstract

**Background:**

High-grade AV block (HAVB) is the most frequent adverse event after transcatheter aortic valve implantation (TAVI). In rare cases, HAVB is associated with hemodynamic compromise (HC) followed by syncope or application of cardiopulmonary resuscitation (CPR), but data on this severe complication are scarce. The aim of the present study was to investigate the incidence and predictors of HC due to HAVB in patients undergoing TAVI.

**Methods:**

In this retrospective analysis of 4,602 TAVI cases between 2010 and 2022, 466 developed HAVB. Baseline characteristics and procedural and postprocedural findings were compared for patients with HC versus those without. Univariate and multivariable regression analyses were used to investigate independent predictors of HC.

**Results:**

Forty-nine of 466 patients (10.5%) had HC due to HAVB after TAVI. Patients with HC had a longer hospital stay [10 (8–13) vs. 13 (9–18) days; *p* < 0.001], more frequent peripheral artery disease (PAD) (28.6% vs. 15.1%; *p* = 0.016), and lower hemoglobin levels [11.8 (±) vs. 12.5 (±) g/dl; *p* = 0.006]. In the HC group, HAVB onset post-TAVI was delayed compared with the non-HC group [2 (1–4) vs. 1 (0–3) days; *p* < 0.001]. Before HAVB onset, patients in the HC group more frequently developed post-TAVI delirium [18 (4.6%) vs. 11 (25.0%); *p* < 0.001]. In univariate regression analysis, PAD, hemoglobin, procedural time, contrast agent volume, and post-TAVI delirium were significant predictors of HC. After adjustment, only post-TAVI delirium and contrast agent volume remained independent predictors [OR 3.22 (95% CI: 1.05–9.89); *p* = 0.042 and OR: 1.01 (95% CI: 1.0–1.01); *p* = 0.04, respectively].

**Conclusion:**

HC due to HAVB after TAVI occurred in over 10% of cases. Development of post-TAVI delirium and contrast agent volume are independent predictors of this severe complication.

## Introduction

Transcatheter valve implantation (TAVI) has become the first-line treatment modality for patients with symptomatic severe aortic stenosis and at least 75 years of age or patients below 75 years who are unsuitable for surgical valve replacement (SAVR) but suitable for transfemoral TAVI (1). Compared with SAVR, the rates of new permanent pacemaker implantation (PPI), most commonly due to high-grade AV block (HAVB), are consistently higher in patients undergoing TAVI, ranging from approximately 4% up to 10% for balloon-expandable transcatheter heart valves (THV) (2–5), 12%–20% for the self-expanding Evolut R/Pro (Medtronic, Minneapolis, MN, USA) THV (6–8, 5), and up to 10% for the self-expanding Acurate Neo THV (Boston Scientific, Marlborough, MA, USA) (9).

Right ventricular pacing after PPI leads to inter- and intraventricular dyssynchrony, causing left ventricular (LV) remodeling (10), mitral regurgitation (11), and impaired LV ejection fraction (LVEF) (12). These adverse effects contribute to the higher rates of heart failure, recurrent hospitalizations, and poorer recovery of LV function described for TAVI patients with new PPI (13). Conversely, cardiac death rates are lower in TAVI patients with new PPI, most likely due to the protection from high-grade conduction disturbances and subsequent sudden cardiac death afforded by a pacemaker (14). Thus, advantages and disadvantages of a planned PPI in TAVI patients should be carefully considered, and knowledge of potential predictors of adverse events due HAVB can be helpful in guiding decisions.

The aim of the current study was to investigate the incidence of HAVB with and without hemodynamic compromise (HC) followed by cardiopulmonary resuscitation (CPR) or syncope in TAVI patients, identify predictors of HC, and analyze potential differences in baseline, procedural, and postprocedural characteristics between patients with and without HC due to HAVB.

## Methods

### Patient population

**Figure 1:**
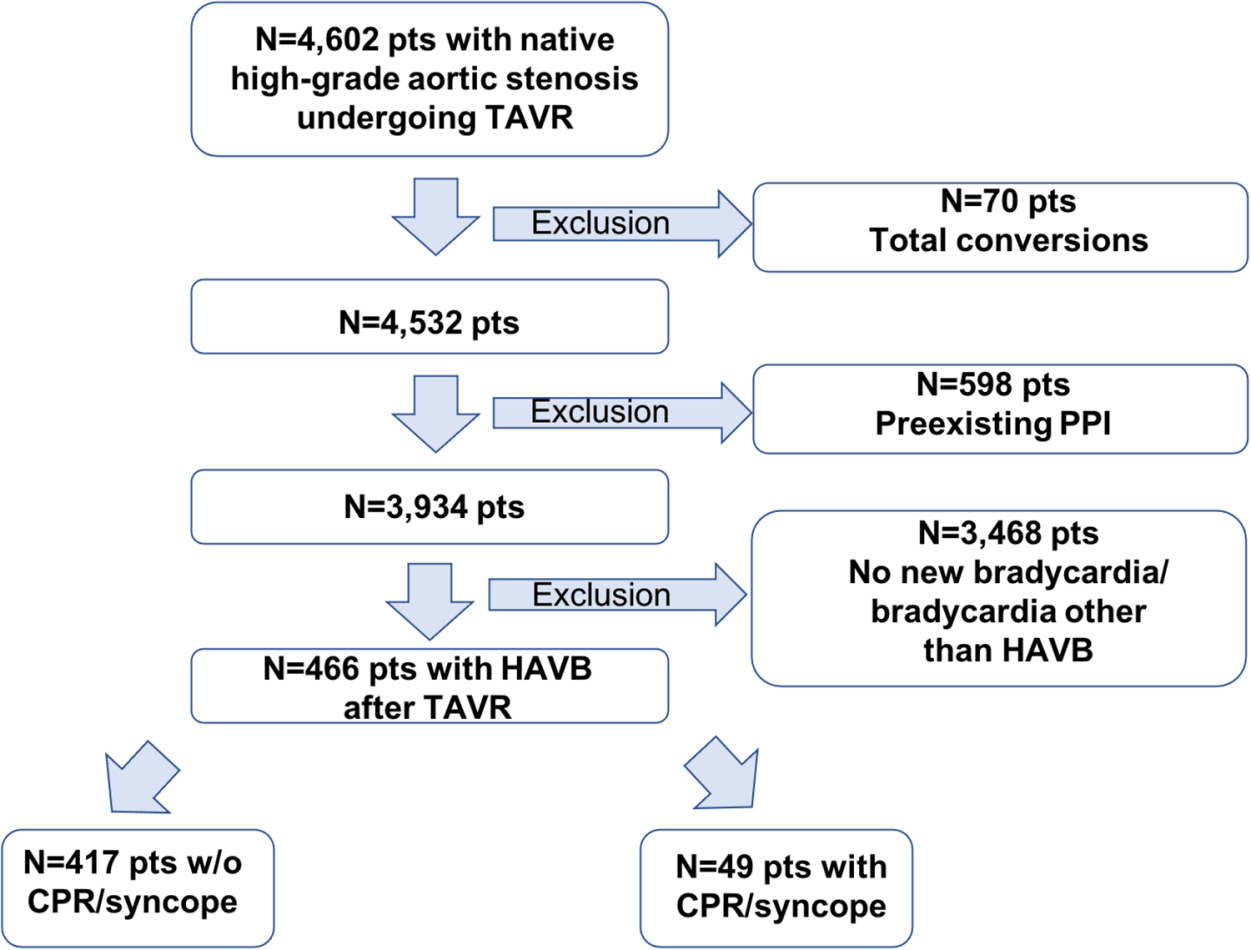
Flowchart of the overall cohort. PPI, permanent pacemaker implantation; HAVB, high-grade atrio-ventricular block; CPR, cardiopulmonary resuscitation.

Between January 2010 and February 2022, 4,602 consecutive patients with symptomatic severe native aortic stenosis underwent TAVI in our institution. Patients were found to be eligible for TAVI based on the clinical consensus of a multidisciplinary heart team consisting of interventional cardiologists, cardiothoracic surgeons, and anesthesiologists. Patients with conversions to open heart surgery (*n* = 70), preexisting PPI (*n* = 598), and no new bradycardia or bradycardia other than AV block grade II type 2 or grade III after TAVI (*n* = 3,468) were excluded from analysis. The final study cohort comprised 466 patients (Figure 1). The medical charts were reviewed to identify the onset of HAVB and HAVB-associated cardiac adverse events (CPR and syncope), bradycardia-inducing medication, the presence of a transvenous pacer at the time of the onset of HAVB, and development of post-TAVI delirium before the onset of HAVB. Patients with documented CPR or syncope due to HAVB were assigned to the group HAVB with HC; patients with documented HAVB but without the need for CPR or syncope were assigned to the group HAVB without HC. Procedural outcomes and complications were defined according to the Valve Academic Research Consortium (VARC) III criteria (15). The decision regarding PPI was made in consensus between the TAVI operator and the electrophysiologist according to guideline recommendations (16). Follow-up data were obtained via outpatient visits, telephone interview, or medical reports from referring hospitals/general practitioners. Follow-up data on 30-day outcome was available for all patients. The study was conducted in adherence to the Declaration of Helsinki and was approved by the Ethics Committee of the University of Giessen, Giessen, Germany (AZ 180/20).

### Statistical methods

Continuous variables are presented as mean with standard deviation (SD) or as median with interquartile range (IQR), as appropriate. Categorical variables are given as frequencies and percentages. The presence of a normal distribution pattern was tested using the Shapiro–Wilk test. The Mann–Whitney-*U* test was used for comparison of continuous variables when not normally distributed, and the unpaired *t*-test was applied when normal distribution was present. For comparison of categorical variables, the *χ*^2^ test was applied. Binary logistic univariate regression analysis with “HC” as dependent variable was performed for baseline variables including, age, sex, body mass index (BMI), hemoglobin (Hb) levels, chronic renal insufficiency (defined by eGFR <60 ml/min/1.73 m^2^), EuroScore II, baseline conduction disturbances (first-degree AV block, left bundle branch block (LBBB), right bundle branch block (RBBB)), NYHA class >2, and history of syncope as well as for pre-TAVI computed tomographic (CT) measures of the aortic root, the presence of LV outflow tract (LVOT) calcification, the length of membranous septum, and baseline echocardiographic parameters and for clinical parameters including cardiovascular risk factors and preexisting cardiovascular diseases. Furthermore, procedural/postprocedural factors including transapical access, predilatation, postdilatation, implantation depth, procedural time and contrast volume, vascular complications, delirium, and postprocedural echocardiographic findings were included. All variables with a *p*-value <0.1 were included in the multivariable analysis. Significance was assumed when a two-sided *p*-value <0.05 was determined. SPSS Version 22.0 (IBM, Armonk, New York, USA) was used for all statistical analyses.

## Results

### Patient characteristics

A total of 466 patients (50% female) with a median age of 82 (79–85) years were included in the analysis. After TAVI, 417 patients (89.5%) developed HAVB without HC whereas 49 patients (10.5%) had HC due to HAVB. In the HC group, 75% had CPR and 25% experienced syncope due to HAVB. Table 1 depicts the baseline characteristics of the entire cohort and both subgroups (HAVB without HC and HAVB with HC). There were no differences in demographic parameters, cardiovascular risk factors, or preexisting cardiac diseases between the groups. Patients who had HAVB with HC had a longer hospital stay [13 (9–18) days vs. 10 (8–13) days; *p* < 0.001]. Hb was lower in the HC group (12.5 ± 1.8 vs. 11.8 ± 1.6 g/dl; *p* = 0.006), and peripheral artery disease (PAD) was more frequent in this group (28.6% vs. 15.1%; *p* = 0.016). No differences in baseline conduction abnormalities according to 12-lead electrocardiogram (ECG) on admission and baseline echocardiography findings were observed between the groups. Anatomic measures of the aortic root according to pre-TAVI CT were comparable (Table 1).

**Table 1:** Baseline characteristics.

	Total cohort (*n* = 466)	HAVB without HC (*n* = 417)	HAVB with HC (*n* = 49)	*p*-value
Age, years	82 (79–85)	82 (79–85)	82 (78–87)	0.92
Sex, male	233 (50.0)	210 (50.4)	23 (46.9)	0.651
Body mass index, kg/m^2^	27.1 (24.3–31.0)	27.1 (24.2–31.1)	27.1 (24.7–30.8)	0.881
Hb, g/dl	12.5 (±)	12.5 (±)	11.8 (±)	***0*** ***.*** ***006***
NYHA class 3 or 4	366 (78.5)	327 (78.4)	39 (79.6)	0.85
Prior syncope	62 (13.3)	52 (12.5)	10 (20.4)	0.624
Prior cardiac decompensation	159 (34.1)	139 (33.3)	20 (40.8)	0.296
Hypertension	420 (90.1)	375 (89.9)	45 (91.8)	0.672
Diabetes mellitus	162 (34.8)	140 (33.6)	22 (44.9)	0.115
Hyperlipidemia	195 (41.8)	171 (41.0)	24 (49.0)	0.285
EuroScore II, %	3.1 (2.1–5.1)	3.1 (2.1–5.0)	3.4 (2.5–5.8)	0.096
Coronary artery disease	282 (60.5)	250 (60.0)	32 (65.3)	0.468
Previous MI	58 (12.4)	54 (12.9)	4 (8.2)	0.337
Peripheral artery disease	77 (16.5)	63 (15.1)	14 (28.6)	***0*** ***.*** ***016***
Chronic renal insufficiency (eGFR <60 ml/min/1.73 m^2^)	216 (46.8)	190 (46.0)	26 (53.1)	0.349
COPD	98 (21.0)	89 (21.3)	9 (18.4)	0.629
Prior stroke	59 (12.7)	51 (12.2)	8 (16.3)	0.415
Atrial fibrillation	172 (36.9)	154 (36.9)	18 (36.7)	0.979
First-degree AVB	138 (29.6)	125 (30.0)	13 (26.5)	0.617
RBBB	147 (31.5)	136 (32.6)	11 (22.4)	0.147
LBBB	38 (8.2)	32 (7.7)	6 (12.2)	0.269
Prior CABG	54 (11.6)	46 (11.0)	8 (16.3)	0.273
Ejection fraction, %	655 (55–65)	65 (55–65)	65 (60–65)	0.405
Pmean, mmHg	44 (36–54)	45 (36–55)	44 (36–51)	0.465
Aortic valve area, cm^2^	0.7 (0.6–0.8)	0.7 (0.5–0.8)	0.7 (0.6–0.8)	0.42
Length of hospital stay, days	10 (8–14)	10 (8–13)	13 (9–18)	***<0*** ***.*** ***001***
Pre-TAVI CT findings				
Area-derived annulus diameter, mm^2^	24.2 (2.2)	24.2 (2.2)	24.0 (2.2)	0.494
Perimeter-derived annulus diameter, mm	24.8 (2.2)	24.8 (2.2)	24.6 (2.2)	0.511
Cover index, perimeter, %	5.2 (2.3–8.0)	5.1 (2.3–7.9)	6.0 (2.3–8.4)	0.46
Cover index, area, %	7.4 (4.3–10.4)	7.3 (4.1–10.4)	7.5 (4.5–10.9)	0.46
LVOT diameter, mm	23.3 (2.8)	23.4 (2.8)	22.8 (2.8)	0.191
SOV diameter, mm	32.2 (3.5)	32.2 (3.5)	32.0 (3.4)	0.653
STJ diameter, mm	28.5 (26.6–30.5)	28.5 (26.6–30.5)	28.0 (26.6–31.0)	0.75
Aorto-annular angle,°	51.2 (8.9)	51.3 (9.0)	50.4 (8.0)	0.67
Bicuspid valve	45 (9.7)	38 (9.1)	7 (14.3)	0.246
Aortic valve calcium score, AU	2,843 (1,900–3,920)	2,862 (1,900–3,934)	2,492 (1,891–4,189)	0.698
Membranous septum length, mm	4.0 (2.5–6.0)	4.0 (2.5–6.0)	4.0 (∼3.0–5.0)	0.66
LVOT calcification	92 (24.2)	80 (23.2)	12 (34.3)	0.144

Baseline demographic, clinical, electrocardiographic, echocardiographic, and CT characteristics of the total cohort and the subgroups HAVB without HC and *HAVB* with HC. Continuous variables are depicted as median with interquartile range or as mean with standard deviation (SD) as appropriate. Categorical parameters are presented as numbers with percentages.

HR, hemodynamic compromise; HAVB, high-grade atrioventricular block; NYHA, New York heart association; MI, myocardial infarction; eGFR, estimated glomerular filtration rate; COPD, chronic obstructive pulmonary disease; RBBB, right bundle branch block; LBBB, left bundle branch block; CABG, coronary artery bypass graft; Pmean, mean transvalvular aortic gradient; LVOT, left ventricular outflow tract, SOV, sinus of valsalva; STJ, sinotubular junction; AU, agatston unit.

### Procedural and postprocedural findings

The transapical access route was more often used in the HC group than in the group without HC (30.6% vs. 13.2%; *p* = 0.003). No significant differences were observed for the rate of predilatation, postdilatation, THV type, fluoroscopy time, procedural time, and implantation depth, although there was a tendency for a longer procedural time in the group of patients with HC [49 (28–64) vs. 40 (32–52); *p* = 0.215]. Postprocedural echocardiographic findings did not differ between the groups. Patients in the HC group had more often type 2–4 bleeding and they suffered more frequently from acute kidney injury (AKI)  stage 2 and more often experienced stroke events. Device success was more often achieved in the group without HC. Table 2 displays the procedural and postprocedural findings for the entire cohort and both subgroups.

**Table 2:** Procedural and postprocedural outcomes.

	Total cohort (*n* = 466)	HAVB without HC (*n* = 417)	HAVB with HC (*n* = 49)	*p*-value
Access				*0* *.* *003*
Transfemoral	392 (84.1)	359 (86.1)	33 (67.3)	* *
Transapical	70 (15)	55 (13.2)	15 (30.6)	* *
Other (transaortal/transsubclavian)	4 (0.9)	3 (0.7)	1 (2)	* *
Prosthesis type				0.765
Balloon-expandable^a^	179 (38.5)	159 (38.2)	20 (40.8)	
Self-expanding, CoreValve type^b^	108 (23.2)	98 (23.6)	10 (20.4)	
Self-expanding, ACURATE type^c^	171 (36.8)	152 (36.5)	19 (38.8.0)	
Other^d^	7 (1.5)	7 (1.7)	0 (0)	
Procedural characteristics				
Predilatation	277 (59.4)	244 (58.5)	33 (67.3)	0.234
Postdilatation	121 (26.0)	108 (25.9)	13 (26.5)	0.924
Implantation depth (NCC), mm	5 (3–7)	5 (3–7)	6 (4–7)	0.208
Procedural time, min	40 (32–52)	40 (32–52)	49 (28–64)	0.215
Contrast agent volume, ml	80 (50–110)	80 (50–110)	70 (45–140)	0.943
Procedural and postprocedural complications				
Bleeding, type 2–4	129 (27.9)	97 (23.4)	32 (65.3)	***<0*** ***.*** ***001***
Major vascular complications	38 (8.2)	32 (7.7)	6 (12.2)	0.269
Device success	349 (74.9)	323 (77.5)	26 (53.1)	***<0*** ***.*** ***001***
Stroke (NeurARC 1a-d)	23 (4.9)	16 (3.8)	7 (14.3)	***0*** ***.*** ***001***
AKI stage 2	58 (12.4)	47 (11.3)	11 (22.4)	***0*** ***.*** ***025***
Postprocedural echocardiographic findings				
Left ventricular ejection fraction, %	65 (60–65)	65 (60–65)	65 (60–65)	0.855
Pmean, mmHg	10 (7–13)	10 (7–13)	9 (6–13)	0.299
Aortic valve area, cm^2^	1.6 (1.4–1.8)	1.6 (1.4–1.8)	1.5 (1.4–1.9)	0.567
Paravalvular leakage ≥ moderate	25 (5.4)	20 (4.8)	5 (10.2)	0.112
Follow-up				
30-day all-cause mortality	24 (5.2)	12 (2.9)	12 (24.5)	***<0.001***

Comparison of procedural and postprocedural findings of the subgroups “no-HC” and “HC”. Significant values are depicted in italics and bold.

HAVB, high-degree AV block; HC, hemodynamic compromise; NCC, non-coronary cusp; AKI, acute kidney injury, Pmean, mean transvalvular aortic gradient.

^a^ Sapien XT, Sapien 3, Sapien Ultra, Myval.

^b^ CoreValve, EvolutR/Pro, Portico, Portico FN, Hydra, Navitor.

^c^ Symetis TA, Symetis TF, ACURATE Neo TA, ACURATE neo2.

^d^ JenaValve, Engager, Directflow, Lotus.

### HAVB-associated postprocedural findings and differences between subgroups

HAVB occurred markedly later in the HC group compared with patients without HC: 2 (1–4) vs. 1 (0–3) days; *p* < 0.001. The intake of potential bradycardia-inducing medication up to 24 h before the HAVB event was not different between the groups. The incidence of a new delirium episode after TAVI before the onset of HAVB was more frequent in patients with HC: 25% vs. 4.6%; *p* < 0.001. Patients with HAVB without the need for CPR or the occurrence of syncope had more often a transvenous pacer inserted. Table 3 shows the HAVB-associated findings of the entire cohort and both subgroups.

**Table 3:** HAVB-associated clinical differences between patients with HC and without HC.

	HAVB without HC (*n* = 417)	HAVB with HC (*n* = 49)	*p*-value
Onset of HAVB after TAVI, days	1 (0–3)	2 (1–4)	***<0*** ***.*** ***001***
Transvenous pacer^a^	277 (70.8)	20 (44.4)	***<0*** ***.*** ***001***
Bradycardia-inducing medication^b^^,^^c^	244 (62.4)	33 (76.7)	0.063
Post-TAVI delirium before onset of HAVB^d^	18 (4.6)	11 (25.0)	***<0*** ***.*** ***001***
PPI after TAVI, days	3 (1–6)	4 (2–6)	0.068

Continuous variables are depicted as median with interquartile range, categorical parameters are presented as numbers with percentages. Significant values are depicted in bold and italics.

HAVB, high-degree AV block; HR, hemodynamic compromise; PPI, permanent pacemaker implantation.

^a^ Information available in 391 pts without HC and 45 pts with HC.

^b^ Bradycardia-inducing medication defined as beta-blockers, non-dihydropyridine calcium-channel blocker (verapamil, diltiazem), digitalis, amiodarone and other anti-arrhythmic medication within the last 24 h before onset of HAVB.

^c^ Information available in 391 pts without HC and 43 pts with HC.

^d^ Information available in 406 pts without HC and 29 pts with HC.

## 30-day Outcome and predictors of HC due to HAVB

Short-term all-cause mortality was higher in patients with HC compared with those without HC (24.5% vs. 2.9%; *p* < 0.001) (Table 2). In univariate regression analysis, PAD, preprocedural Hb, procedural time, periprocedural contrast agent volume, and delirium before the onset of HAVB were significant predictors of HC. However, in multivariable analysis, only the presence of delirium before the onset of HAVB and contrast agent volume independently predicted HC: 3.22 (95% CI: 1.05–9.89); *p* = 0.042 and 1.01 (95% CI: 1.0–1.01); *p* = 0.04; respectively. Table 4 shows the results of the univariate und multivariable regression analyses.

**Table 4:** Unadjusted and adjusted predictors of HC due to HAVB.

	Univariate OR (95% CI)	*p*-value	Multivariable OR (95% CI)	*p*-value
Baseline characteristics				
Age, years	1.00 (0.95–1.06)	0.919	–	–
Sex, male	0.87 (0.48–1.58)	0.651	–	–
Body mass index, kg/m^2^	1.00 (0.95–1.06)	0.987	–	–
EuroScore II, %	1.03 (0.97–1.10)	0.317	–	–
NYHA class 3 or 4	1.07 (0.52–2.23)	0.85	–	–
Cardiac decompensation	1.38 (0.75–2.53)	0.297	–	–
Syncope	1.80 (0.85–3.82)	0.126	–	–
Arterial hypertension	1.26 (0.43–3.68)	0.672	–	–
Diabetes mellitus	1.62 (0.89–2.93)	0.118	–	–
Coronary artery disease	1.26 (0.68–2.34)	0.469	–	–
Prior MI	0.59 (0.21–1.73)	0.342	–	–
Peripheral artery disease	2.25 (1.14–4.42)	***0*** ***.*** ***019***	1.16 (0.42–3.19)	0.779
CABG	1.57 (0.70–3.56)	0.277	–	–
Chronic renal insufficiency, eGFR < 60 ml/min/1.73 m^2^	0.99 (0.54–1.83)	0.979	–	–
Hb, g/dl	0.80 (0.68–0.94)	***0*** ***.*** ***007***	0.85 (0.70–1.05)	0.135
Baseline ECG parameters			–	–
Atrial fibrillation	0.99 (0.54–1.83)	0.979	–	–
First-degree AV block	0.84 (0.43–1.65)	0.618	–	–
RBBB	0.60 (0.30–1.21)	0.151	–	–
LBBB	1.8 (0.71–4.57)	0.216	–	–
Baseline echocardiographic parameters				
Left ventricular ejection fraction, %	1.01 (0.98–1.04)	0.462	–	–
Pmean, mmHg	0.99 (0.98–1.02)	0.726	–	–
Aortic valve area, mm^2^	2.01 (0.32–12.53)	0.453	–	–
Pre-TAVI CT findings				
Aortic valve calcium score, AU	1.00 (1.00–1.00)	0.93	–	–
Aortic annulus area, mm^2^	0.95 (0.82–1.10)	0.494	–	–
Cover index area, mm^2^	1.03 (0.97–1.09)	0.403	–	–
STJ, mm	1.0 (0.9–1.10)	0.954	–	–
LVOT, mm	0.93 (0.83–1.04)	0.192	–	–
Aorto-annular angle,°	0.99 (0.94–1.04)	0.669	–	–
Membranous septum, mm	0.97 (0.8–1.17)	0.73	–	–
LVOT calcification	1.73 (0.82–3.63)	0.148	–	–
Procedural/post-procedural characteristics				
Transapical access	3.0 (1.55–5.8)	***0*** ***.*** ***001***	0.53 (0.15–1.80)	0.307
Predilatation	1.46 (0.78–2.74)	0.236	–	–
Postdilatation	1.03 (0.53–2.02)	0.924	–	–
Procedural time, min	1.01 (1.00–1.02)	***0*** ***.*** ***035***	1.0 (0.99–1.02)	0.757
Contrast agent volume, ml	1.00 (1.00–1.01)	***0*** ***.*** ***076***	1.01 (1.0–1.01)	***0*** ***.*** ***040***
Implant depth (NCC), mm	1.06 (0.96–1.16)	0.262	–	–
Major vascular complications	1.68 (0.66–4.24)	0.273	–	–
Paravalvular leakage ≥ moderate	2.26 (0.81–6.31)	0.121	–	–
Delirium before onset of HAVB	6.91 (3.01–15.85)	***<0*** ***.*** ***001***	3.22 (1.05–9.89)	***0*** *.* ***042***
Postprocedural echocardiographic findings				
Left ventricular ejection fraction, %	1.02 (0.98–1.05)	0.389	–	–
Pmean, mmHg	0.97 (0.91–1.05)	0.475	–	–
Aortic valve area, mm^2^	0.9 (0.28–2.88)	0.859	–	–

Univariate and multivariable regression analysis of baseline clinical factors, procedural and postprocedural variables for analysis of potential predictors of HC due to HAVB. All variables with a *p*-value <0.1 were included in the multivariable regression analysis. Significant values are depicted in bold and italics.

## Discussion

The main results of our study are that (1) HC due to HAVB post-TAVI occurred in 1% (49 of 4,606 patients) of the entire TAVI cohort and in 10.5% of all patients developing HAVB after TAVI; (2) patients with HC due to HAVB had more frequently a delayed onset of HAVB compared with HAVB patients without HC; (3) the development of delirium after TAVI and contrast agent volume were the sole independent predictors of HC due to HAVB.

To the best of our knowledge this is the first study to investigate the incidence and potential predictors of HC due to HAVB in TAVI patients. While baseline characteristics such as RBBB and first-degree AV block have been clearly identified as predictors of the development of HAVB and the subsequent need for PPI after TAVI (17, 18), little is known about the risk of HC when HAVB occurs. Many studies have investigated the incidence and onset of conduction disturbances post-TAVI, but data on the incidence of CPR or syncope associated with HAVB are lacking (19–21). Importantly, this subset of patients may be highly vulnerable and have much worse prognosis, as shown by our finding that the 30-day mortality rate was more than eight times higher in this group than in HAVB patients without HC.

Since its first description in 2002 (22), TAVI has become a routine procedure performed on a daily basis worldwide. During these two decades, alongside procedure-related and THV-related optimizations, vast efforts were undertaken to shorten the index hospital length of stay (23). So called “fast-track pathway” discharges for TAVI patients have become common and have led to a further decrease in the length of hospital stays (24, 25). Just recently, partly due to the coronavirus disease (COVID)-19 pandemic, the feasibility of “same-day” discharge of TAVI patients was demonstrated in several studies (26–28). In all of the abovementioned studies, early discharges had no negative effect on 30-day outcome measures such as cardiovascular mortality or the rate of hospital readmissions. However, as cardiac arrest due to HAVB is a very rare event and most studies investigating the feasibility of early discharge had rather small sample sizes, this specific event may not have been captured. Barker et al. recently demonstrated in the multicenter PROTECT-TAVR study comprising 124 TAVI patients that the same-day discharge concept is feasible with a low cardiovascular hospital readmission rate of 2.8% during the first 30 days; no patient died or required PPI during that period (26). Of note, patients with preexisting RBBB or baseline second-degree type 2 or third-degree AV block were excluded, whereas inclusion of patients with preexisting first-degree or second-degree type I AV block was possible and left to the discretion of the individual sites (26). In our study, the rate of baseline complete RBBB and first-degree AV block was rather high, with an incidence of 31.5% and 29.6%, respectively, without a significant difference between patients with HC and without HC. In our cohort, 50.6% of patients had either first-degree AV block or RBBB. Since both conduction abnormalities are known predictors of HAVB, this is not surprising as only patients with HAVB after TAVI were included in the analysis. On the other hand, approximately 50% of patients had no relevant baseline conduction abnormalities before TAVI and nonetheless developed HAVB afterwards. Naturally, when HAVB is recognized, early discharge is unlikely and results in prolonged monitoring with eventual PPI in most of the cases. However, HAVB might only be transient after TAVI, so that the benefits of PPI must be carefully weighed against potential risks. In a large meta-analysis by Zito et al. comprising over 50,000 TAVI patients, long-term all-cause mortality and rehospitalization rates for heart failure were significantly higher in patients receiving PPI after TAVI during a mean follow-up time of 22 months (29).

In the present study, the vast majority of HAVB episodes (approximately 90%) occurred within a median time of 1 day without HC, which basically supports the early discharge concept. However, although CPR/syncope occurred rarely (1% of all TAVI procedures from 2010 to 2022), HAVB with HC arose with a delay of a median time of 2 days in our study, possibly occurring out of hospital in accordance with the early discharge concept as mentioned above. Of course, early discharge harbors certain advantages, such as the patient’s convenience, cost savings, and sparing of hospital inpatient capacities, which was especially a factor in the time of the COVID-19 pandemic. The evaluation of potential predictors of adverse events such as CPR due to HAVB is thus of utmost importance to guide the decision on early discharge, and the present study is the first to address this issue.

HC due to HAVB is the most serious complication that can occur in the setting of a conduction disturbance. In this situation, either no ventricular escape rhythm or an insufficient rhythm is present, leading to the need for cardiac resuscitation or at least resulting in syncope. In our study, however, nearly 90% of HAVB incidents occurred without the need for CPR or in the absence of significant HC, most likely because in these cases the AV block was only transient, or, if persistent, the patient had sufficient cardiac output despite ventricular escape rhythm. Another reason for the absence of HC could be the higher rate of transvenous pacer inserted in these HAVB patients compared with patients with HC: 70.8% vs. 40.8%; *p* < 0.001. However, in both groups, transvenous pacing did not always work properly since patients in the HC group also developed HC due to HAVB despite an inserted transvenous pacer.

Post-TAVI delirium was found to be an independent predictor of HC due to HAVB. This finding is interesting, since it has never been directly described in the context of HAVB. Nevertheless, it is is known that delirium in TAVI patients is associated with a higher mortality (30–32). In a recently published meta-analysis, Tilley et al. found an incidence of post-TAVI delirium of nearly one quarter of patients examined (33). Transapical access was one of the strongest predictors of delirium [OR 4.0 (95% CI: 2.3–9.9); *p* < 0.001], a finding that is consistent with several other studies investigating delirium in post-TAVI patients (34, 31). The transapical access route was also more frequently used in our HC subgroup. The reasons for this association are diverse: patients undergoing TAVI via the transapical access route require general anesthesia, which is generally associated with a higher postprocedural rate of delirious states compared with rates for local anesthesia (35), which can usually be applied during a transfemoral TAVI approach. Also, transapical access is only used when transfemoral access is not feasible, most likely due to more severe peripheral artery disease, which also might reflect a higher comorbidity burden that in turn is associated with a higher incidence of delirium. The EuroScore II was numerically higher in the HC group compared with the no-HC group [3.4 (2.5–5.8) vs. 3.1 (2.1–5.0); *p* = 0.096], possibly reflecting a higher disease burden in the former group, although the difference was not statistically significant (possibly due to the low sample size of the HC group).

Another factor that might contribute to a higher incidence of post-TAVI delirium that was not addressed in our analysis is the presence of low cardiac output syndrome. Study data showed that potentially due to lower cerebral perfusion, low cardiac syndrome is a contributor to the development of a delirious state after cardiac surgery (36). However, although low cardiac output syndrome as a parameter was not specifically addressed in our study, post-procedural LVEF was not different between HC and no-HC-groups and also was not statistically significant in the univariate logistic regression analysis.

Contrast agent volume was also found to be an independent predictor of HC in our study. Higher amounts of contrast media might also be a triggering factor for post-procedural delirium; however, this assumption remains speculative as no clear evidence exists in this regard. It is also possible that this parameter reflects a higher rate of AKI after TAVI. The latter is described as a strong risk factor for post-TAVI delirium in a recently published meta-analysis: patients who developed AKI had a 5-fold higher risk for the development of delirium after TAVI (33). AKI as a parameter was not included in our regression analysis, since from the data it was not clear in which chronological context this parameter stood with regard to HAVB and HC (see also the discussion of limitations, below), meaning we could not distinguish whether AKI developed before or after HC.

In our study, patients with HC had more frequently a delayed onset of HAVB. This explains why fewer patients still had a transvenous pacer inserted, which is usually withdrawn 24 h after TAVI in cases with no high-grade conduction disturbances in accordance with our in-hospital standard operating procedure. The higher rate of delirium post-TAVI could be the reason why the transvenous pacer (still inserted in 40% of cases) did not work properly, most likely due to dislocation of the lead by an agitated, delirious patient. Although this remains speculative, since from the patients’ charts the reasons for a dislocated pacing lead usually could not be recapitulated, this course of events would be the most likely explanation for the higher incidence of HC in patients with delirium. In addition, the delirious state itself might play a role in making patients more vulnerable and potentially lead to a lack of sufficient ventricular rhythm during HAVB via pathways still not fully understood. In that context, it is a matter of fact that mortality rates are remarkably higher in delirious patients (30–32); however, the reasons for this have thus far never been elucidated, and to our knowledge, no study exists that investigates the actual causes of higher mortality rates in this special patient subset.

Nevertheless, although delirium was evidently more frequent in HC patients, 75% of patients in that group had no delirious state post-TAVI and still had HC. Again, the higher disease burden as reflected by EuroScore II might have also played a role here, as these patients might have fewer resources to hemodynamically compensate during HAVB. As procedural factors such as pre- and postdilatation were not different between the groups with and without HC, it seems unlikely that these factors contributed to HC due to HAVB. Also, the length of the membranous septum, a recently discovered predictor of conduction disturbances after TAVI (37), was not significantly different between the two groups. Device success was markedly lower in the HC group than in the no-HC group. Also, although not significantly different, procedural time was numerically longer in the HC group, possibly reflecting a more complex procedure with eventually lower device success rates. This could also be a contributing factor for post-TAVI delirium, but this remains speculative. It is also conceivable that the lower device success rate was associated with more intraprocedural manipulation at the valve level, potentially leading to a more prolonged HAVB with subsequent cardiac arrest/syncope.

Our study has several limitations. It is a single-center analysis, so the results cannot be simply transferred to other centers, and as it is also retrospective, potential unknown confounders cannot be ruled out. The sample size of the HC group, although comprising a long timeframe of over 12 years in a high-volume TAVI center, is quite low, weakening the statistical power of our analysis. Furthermore, the diagnosis of delirium was taken from the patients’ charts and was mainly based on the individual physician’s evaluation of the orientation of the patient and not on objective scores, for example, the Confusion Assessment Method (CAM)-ICU score. Valid chart review regarding HAVB-associated clinical conditions was not possible in about 30 patients due to insufficient documentation or the chart not being available. Because an older version of TAVI CT scans were used from 2010 to 2013, the parameters LVOT calcification and membranous septum length were not available for all patients. Finally, postprocedural stroke, bleeding type 2–4, and AKI stage 2 were not included in the regression analysis, since it was not always clear in which chronological context these parameters stood in relation to the HAVB and HC.

## Conclusion

HC due to HAVB after TAVI is a rare event that occurred in 1% of all patients in our cohort and in 10.5% of patients with HAVB. Post-TAVI delirium and contrast agent volume were found to be the sole independent predictors of this adverse event. Further studies are needed to validate this finding and to elucidate its pathophysiological role in the context of HC due to HAVB in TAVI patients.

## Data Availability

Data cannot be made publicly available for ethical or legal reasons, e.g., public availability would compromise patient confidentiality or participant privacy. Data are available from the Kerckhoff Institutional Data Access for researchers who meet the criteria for access to confidential data. Any requests for data access may be sent to the administration of the Kerckhoff Heart Center via email at info@kerckhoff-klinik.de or by contacting: Kerckhoff Heart and Thore Center, Geschaeftsfuehrung, Benekestrasse 2–8, 61,231 Bad Nauheim. Requests to access the datasets should be directed to info@kerckhoff-klinik.de.

## References

[1] Vahanian A Beyersdorf F Praz F Milojevic M Baldus S Bauersachs J 2021 Esc/EACTS guidelines for the management of valvular heart disease. Eur Heart J. (2022) 43(7):561–632. 10.1093/eurheartj/ehab395 34453165

[2] Mack MJ Leon MB Thourani VH Makkar R Kodali SK Russo M Transcatheter aortic-valve replacement with a balloon-expandable valve in low-risk patients. N Engl J Med. (2019) 380(18):1695–705. 10.1056/NEJMoa1814052 30883058

[3] Nazif TM Cahill TJ Daniels D McCabe JM Reisman M Chakravarty T Real-world experience with the SAPIEN 3 ultra transcatheter heart valve: a propensity-matched analysis from the United States. Circ Cardiovasc Interv. (2021) 14(9):e010543. 10.1161/CIRCINTERVENTIONS.121.010543 34433290

[4] Saia F Gandolfo C Palmerini T Berti S Doshi SN Laine M In-hospital and thirty-day outcomes of the SAPIEN 3 ultra balloon-expandable transcatheter aortic valve: the S3U registry. EuroIntervention. (2020) 15(14):1240–47. 10.4244/EIJ-D-19-00541 31763985

[5] Costa G Saia F Pilgrim T Abdel-Wahab M Garot P Valvo R Transcatheter aortic valve replacement with the latest-iteration self-expanding or balloon-expandable valves: the multicenter OPERA-TAVI registry. JACC Cardiovasc Interv. (2022) 15(23):2398–407. 10.1016/j.jcin.2022.08.057 36121242

[6] Popma JJ Deeb GM Yakubov SJ Mumtaz M Gada H O’Hair D Transcatheter aortic-valve replacement with a self-expanding valve in low-risk patients. N Engl J Med. (2019) 380(18):1706–15. 10.1056/NEJMoa1816885 30883053

[7] Forrest JK Mangi AA Popma JJ Khabbaz K Reardon MJ Kleiman NS Early outcomes with the evolut PRO repositionable self-expanding transcatheter aortic valve with pericardial wrap. JACC Cardiovasc Interv. (2018) 11(2):160–8. 10.1016/j.jcin.2017.10.014 29348010

[8] Forrest JK Kaple RK Tang GHL Yakubov SJ Nazif TM Williams MR Three generations of self-expanding transcatheter aortic valves: a report from the STS/ACC TVT registry. JACC Cardiovasc Interv. (2020) 13(2):170–9. 10.1016/j.jcin.2019.08.035 31973793

[9] Kim WK Mollmann H Liebetrau C Renker M Rolf A Simon P The ACURATE neo transcatheter heart valve: a comprehensive analysis of predictors of procedural outcome. JACC Cardiovasc Interv. (2018) 11(17):1721–9. 10.1016/j.jcin.2018.04.039 29803694

[10] Prinzen FW Augustijn CH Arts T Allessie MA Reneman RS. Redistribution of myocardial fiber strain and blood flow by asynchronous activation. Am J Physiol. (1990) 259(2 Pt 2):H300–8. 10.1152/ajpheart.1990.259.2.H300 2386214

[11] Kanzaki H Bazaz R Schwartzman D Dohi K Sade LE Gorcsan J 3rd. A mechanism for immediate reduction in mitral regurgitation after cardiac resynchronization therapy: insights from mechanical activation strain mapping. J Am Coll Cardiol. (2004) 44(8):1619–25. 10.1016/j.jacc.2004.07.036 15489094

[12] Nahlawi M Waligora M Spies SM Bonow RO Kadish AH Goldberger JJ. Left ventricular function during and after right ventricular pacing. J Am Coll Cardiol. (2004) 44(9):1883–8. 10.1016/j.jacc.2004.06.074 15519023

[13] Chamandi C Barbanti M Munoz-Garcia A Latib A Nombela-Franco L Gutierrez-Ibanez E Long-term outcomes in patients with new permanent pacemaker implantation following transcatheter aortic valve replacement. JACC Cardiovasc Interv. (2018) 11(3):301–10. 10.1016/j.jcin.2017.10.032 29413244

[14] Regueiro A Abdul-Jawad Altisent O Del Trigo M Campelo-Parada F Puri R Urena M Impact of new-onset left bundle branch block and periprocedural permanent pacemaker implantation on clinical outcomes in patients undergoing transcatheter aortic valve replacement: a systematic review and meta-analysis. Circ Cardiovasc Interv. (2016) 9(5):e003635. 10.1161/CIRCINTERVENTIONS.115.003635 27169577

[15] Genereux P Piazza N Alu MC Nazif T Hahn RT Pibarot P Valve academic research consortium 3: updated endpoint definitions for aortic valve clinical research. Eur Heart J. (2021) 42(19):1825–57. 10.1093/eurheartj/ehaa799 33871579

[16] Brignole M Auricchio A Baron-Esquivias G Bordachar P Boriani G Breithardt OA 2013 Esc guidelines on cardiac pacing and cardiac resynchronization therapy: the task force on cardiac pacing and resynchronization therapy of the European society of cardiology (ESC). Developed in collaboration with the European heart rhythm association (EHRA). Eur Heart J. (2013) 34(29):2281–329. 10.1093/eurheartj/eht150 23801822

[17] Siontis GC Juni P Pilgrim T Stortecky S Bullesfeld L Meier B Predictors of permanent pacemaker implantation in patients with severe aortic stenosis undergoing TAVR: a meta-analysis. J Am Coll Cardiol. (2014) 64(2):129–40. 10.1016/j.jacc.2014.04.033 25011716

[18] Nazif TM Dizon JM Hahn RT Xu K Babaliaros V Douglas PS Predictors and clinical outcomes of permanent pacemaker implantation after transcatheter aortic valve replacement: the PARTNER (placement of AoRtic TraNscathetER valves) trial and registry. JACC Cardiovasc Interv. (2015) 8(1 Pt A):60–9. 10.1016/j.jcin.2014.07.022 25616819

[19] Gaede L Kim WK Liebetrau C Dorr O Sperzel J Blumenstein J Pacemaker implantation after TAVI: predictors of AV block persistence. Clin Res Cardiol. (2018) 107(1):60–9. 10.1007/s00392-017-1158-2 28963581

[20] Blumenstein J Kim WK Liebetrau C Gaede L Kempfert J Walther T Challenges of coronary angiography and intervention in patients previously treated by TAVI. Clin Res Cardiol. (2015) 104(8):632–9. 10.1007/s00392-015-0824-5 25720330

[21] Poels TT Engels EB Kats S Veenstra L van Ommen V Vernooy K Occurrence and persistency of conduction disturbances during transcatheter aortic valve implantation. Medicina. (2021) 57(7). 10.3390/medicina57070695 PMC8303948 34356976

[22] Cribier A Eltchaninoff H Bash A Borenstein N Tron C Bauer F Percutaneous transcatheter implantation of an aortic valve prosthesis for calcific aortic stenosis: first human case description. Circulation. (2002) 106(24):3006–8. 10.1161/01.cir.0000047200.36165.b8 12473543

[23] Lauck SB Wood DA Baumbusch J Kwon JY Stub D Achtem L Vancouver transcatheter aortic valve replacement clinical pathway: minimalist approach, standardized care, and discharge criteria to reduce length of stay. Circ Cardiovasc Qual Outcomes. (2016) 9:312–21. 10.1161/CIRCOUTCOMES.115.002541 27116975

[24] Marcantuono R Gutsche J Burke-Julien M Anwaruddin S Augoustides JG Jones D Rationale, development, implementation, and initial results of a fast track protocol for transfemoral transcatheter aortic valve replacement (TAVR). Catheter Cardiovasc Interv. (2015) 85(4):648–54. 10.1002/ccd.25749 25413312

[25] Durand E Eltchaninoff H Canville A Bouhzam N Godin M Tron C Feasibility and safety of early discharge after transfemoral transcatheter aortic valve implantation with the edwards SAPIEN-XT prosthesis. Am J Cardiol. (2015) 115(8):1116–22. 10.1016/j.amjcard.2015.01.546 25726383

[26] Barker M Sathananthan J Perdoncin E Devireddy C Keegan P Grubb K Same-day discharge post-transcatheter aortic valve replacement during the COVID-19 pandemic: the multicenter PROTECT TAVR study. JACC Cardiovasc Interv. (2022) 15(6):590–8. 10.1016/j.jcin.2021.12.046 35331450 PMC8936029

[27] Perdoncin E Greenbaum AB Grubb KJ Babaliaros VC Keegan P Ceretto-Clark B Safety of same-day discharge after uncomplicated, minimalist transcatheter aortic valve replacement in the COVID-19 era. Catheter Cardiovasc Interv. (2021) 97(5):940–7. 10.1002/ccd.29453 33382519

[28] Krishnaswamy A Isogai T Agrawal A Shekhar S Puri R Reed GW Feasibility and safety of same-day discharge following transfemoral transcatheter aortic valve replacement. JACC Cardiovasc Interv. (2022) 15(6):575–89. 10.1016/j.jcin.2022.01.013 35331449

[29] Zito A Princi G Lombardi M D'Amario D Vergallo R Aurigemma C Long-term clinical impact of permanent pacemaker implantation in patients undergoing transcatheter aortic valve implantation: a systematic review and meta-analysis. Europace. (2022) 24(7):1127–36. 10.1093/europace/euac008 35138367 PMC9460982

[30] Goudzwaard JA de Ronde-Tillmans M de Jager TAJ Lenzen MJ Nuis RJ van Mieghem NM Incidence, determinants and consequences of delirium in older patients after transcatheter aortic valve implantation. Age Ageing. (2020) 49(3):389–94. 10.1093/ageing/afaa001 32091096 PMC7577406

[31] Huded CP Huded JM Sweis RN Ricciardi MJ Malaisrie SC Davidson CJ The impact of delirium on healthcare utilization and survival after transcatheter aortic valve replacement. Catheter Cardiovasc Interv. (2017) 89(7):1286–91. 10.1002/ccd.26776 27566989

[32] Eide LS Ranhoff AH Fridlund B Haaverstad R Hufthammer KO Kuiper KK Readmissions and mortality in delirious versus non-delirious octogenarian patients after aortic valve therapy: a prospective cohort study. BMJ Open. (2016) 6(10):e012683. 10.1136/bmjopen-2016-012683 27707832 PMC5073576

[33] Tilley E Psaltis PJ Loetscher T Davis DH Harrison SL Kim S Meta-analysis of prevalence and risk factors for delirium after transcatheter aortic valve implantation. Am J Cardiol. (2018) 122(11):1917–23. 10.1016/j.amjcard.2018.08.037 30293651 PMC6269593

[34] Maniar HS Lindman BR Escallier K Avidan M Novak E Melby SJ Delirium after surgical and transcatheter aortic valve replacement is associated with increased mortality. J Thorac Cardiovasc Surg. (2016) 151(3):815–23 e812. 10.1016/j.jtcvs.2015.10.114 26774165 PMC5088104

[35] Li T Dong T Cui Y Meng X Dai Z. Effect of regional anesthesia on the postoperative delirium: a systematic review and meta-analysis of randomized controlled trials. Front Surg. (2022) 9:937293. 10.3389/fsurg.2022.937293 35959124 PMC9360531

[36] Norkiene I Ringaitiene D Kuzminskaite V Sipylaite J. Incidence and risk factors of early delirium after cardiac surgery. Biomed Res Int. (2013) 2013:323491. 10.1155/2013/323491 24102052 PMC3786514

[37] Hamdan A Guetta V Klempfner R Konen E Raanani E Glikson M Inverse relationship between membranous septal length and the risk of atrioventricular block in patients undergoing transcatheter aortic valve implantation. JACC Cardiovasc Interv. (2015) 8(9):1218–28. 10.1016/j.jcin.2015.05.010 26292585

